# Efficiency of fluralaner pour-on in different strategic control protocols against *Rhipicephalus microplus* on Brangus cattle in a tropical area

**DOI:** 10.1186/s13071-024-06199-4

**Published:** 2024-03-06

**Authors:** Guilherme Henrique Reckziegel, Mariana Green de Freitas, Juliane Francielle Tutija, Vinícius Duarte Rodrigues, Dyego Gonçalves Lino Borges, Murilo Damasceno Brunet de Freitas, Tiago Gallina, Welber Daniel Zanetti Lopes, Daniel de Castro Rodrigues, Heitor de Oliveira Arriero Amaral, Tom Strydom, Siddhartha Torres, Fernando de Almeida Borges

**Affiliations:** 1 Federal University of Mato Grosso do Sul, Campo Grande, Mato Grosso do Sul Brazil; 2 Federal University of Acre, Rio Branco, Acre Brazil; 3 Federal University of Pampa, Uruguaiana, Brazil; 4 Centro de Parasitologia Veterinária, Universidade Federal de Goiás, Goiânia, Goiás Brazil; 5 MSD Animal Health, São Paulo, Brazil; 6 MSD Animal Health, 20 Spartan Road, Kempton Park, Isando, 1619 South Africa; 7 MSD Animal Health, 2 Giralda Farms, Madison, NJ 07940 USA

**Keywords:** Bovine, Tick, Isoxazolines

## Abstract

**Background:**

The occurrence of higher winter temperatures in Brazilian areas with tropical and highland climates may result in a fifth peak of tick populations during winter in addition to the four generations previously described. Therefore, a strategic control protocol was developed with treatments in two seasons with the objective of controlling the generations of ticks that occur in spring/summer and those that occur in autumn/winter.

**Methods:**

The study was conducted in Mato Grosso do Sul, Brazil, from the beginning of the rainy season, November 2020, to October 2021. In a randomized block design, 36 calves were distributed into three groups: (i) negative control; (ii) traditional strategic control in one season (SC1S), at the beginning of the rainy season; and (iii) strategic control in two seasons (SC2S), at the beginning and end of the rainy season. The SC1S strategic control group was treated on day 0, November 2020, and twice more with intervals of 42 days. The SC2S group received three more treatments beginning on day 182, May 2021, with intervals of 42 days. All treatments consisted of 5% fluralaner (Exzolt^®^ 5%) delivered via a pour-on dose of 1 mL/20 kg body weight. Counts of semi-engorged female ticks were performed on day 3 and every 14 days thereafter, and the animals were weighed at the same time.

**Results:**

Fluralaner showed a mean efficacy of more than 95% up to day 294. The two treated groups showed a decrease (*P* < 0.05) in the average number of ticks on day 3. In the SC2S group, the means were close or equal to zero throughout the study, while in the SC1S group, the means did not differ (*P* > 0.05) from those of the control group from day 231 onward. The final mean weight gain of each group was 76.40 kg, 98.63 kg, and 115.38 kg for the control, SC1S, and SC2S groups, respectively, differing (*P* < 0.05) from each other.

**Conclusions:**

Therefore, three applications of fluralaner, with one application every 42 days from the beginning of the rainy season in the middle spring, resulted in effective tick control for 224 days. When three additional treatments were given in autumn/winter with intervals of 42 days between applications, tick counts were reduced throughout the year. This strategic control approach may be indicated in years with climatic conditions that allow that population peaks are expected to occur in the autumn/winter period.

**Graphical Abstract:**

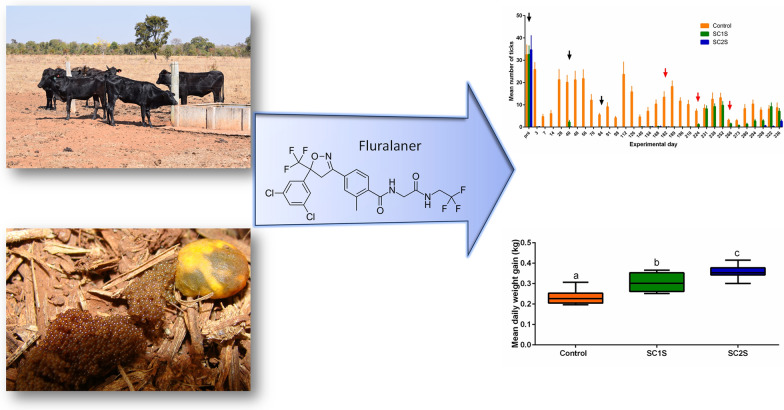

## Background

*Rhipicephalus microplus* [1] is among the major ectoparasites in cattle in tropical and subtropical regions worldwide [2–5]. In Brazil, direct economic losses from *Rhipicephalus* spp., such as decreased weight gain and decreased milk production, generate an estimated impact of US$3.24 billion [6].

Tick control is mainly performed with the use of acaricides, and due to their intense use, the number of reports of acaricide resistance is increasing, and tick strains resistant to multiple drugs have been reported [4, 7–9]. Fluralaner, a molecule belonging to the pharmaceutical group of isoxazolines that is widely used for the control of mites and insects in companion animals, has a long period of action, rapid absorption, and broad spectrum [10] and is a new alternative for the control of *Rhipicephalus* spp. in cattle [11]. To maximize the results and delay resistance, this molecule should be used sustainably and, based on data from field studies and strategic control protocols, allowing for effective control of ticks with minimal use of the chemical.

In countries with rainy summers and dry winters, the occurrence of three or four generations of ticks per year has been reported [12–14]. The infestation peak that occurs shortly after winter [15], with the onset of increased temperatures and increased rainfall in early spring, is considered the first generation. The hotter and wetter summer months allow one or two peaks to occur quickly, thus increasing the number of individuals, leading to the occurrence of another infestation peak during autumn. The milder temperatures and even the presence of humidity in autumn are favorable to *R. microplus* because they prevent the drying of eggs and larvae in the nonparasitic stage [12, 13, 16].

Based on these annual population dynamics, strategic control protocols referred to as winter–spring strategic control were developed. This strategy consists of applications of acaricides from late winter to mid-spring, with the objective of acting before the first infestation peak, leading to the flattening of the infestation curve during the summer and autumn, which have favorable climatic conditions for *R. microplus* [17, 18].

Recent studies have shown that, due to the increase in average temperatures during the winter period, a fifth infestation peak may occur during the coldest season [19, 20]. Thus, the present study aims to evaluate a strategic control protocol, namely, winter–spring and autumn–winter strategic control, with treatments in two seasons, winter–spring and autumn–winter, using fluralaner 5% and compare the results with those obtained with a traditional winter–spring strategic control protocol [17, 18].

## Methods

### Study location and period

The experiment was conducted on a farm that breeds cattle for experimental purposes (20°0′47.86″ S, 53°39′7.45″ W), located in the municipality of Ribas do Rio Pardo, Mato Grosso do Sul. The study area has a tropical semi-humid climate (AW) according to the Köppen–Geiger classification, and the mean temperature of the coldest month is between 18 °C and 20 °C.

The experimental area consisted of three paddocks of 10 hectares with mixed formations of *Brachiaria decumbens* and *B. humidicola*. Each one housed 12 animals, with a stocking rate at the beginning of the study of approximately 0.44 AU/Ha and of 0.65, 0.71 and 0.78 AU/Ha at the end of the study for control, strategic control in one season (SC1S), and strategic control in two seasons (SC2S), respectively.

Three months before the beginning of the study, 36 Nelore × Angus calves aged 8–10 months and naturally infested by *R. microplus* were introduced into the experimental area, and all animals had free access to the three paddocks to ensure that populations of *R. microplus* were present in the environment.

The study began in November 2020 and ended in October 2021, lasting 336 days.

### Experimental design

The animals were distributed in a randomized block design based on weight and tick burden on day 0 into three groups (*n* = 12): (I) control; (II) traditional strategic control in one season, at the beginning of the rainy season, called “SC1S”; and (III) new strategic control in two seasons, at beginning and end of the rainy season, called “SC2S.”

Each group occupied an exclusive paddock throughout the study, which was randomly designated.

### Treatments

All treatments were performed with a formulation of 5% fluralaner (Exzolt^®^ 5%—MSD Saúde Animal), administered via pour-on treatment at a dose of 1 mL/20 kg and exhibiting efficacy above 95% between the 7th and 49th day after treatment. This product was chosen owing to the need to use an acaricide with proven efficacy against ticks [11] and with no previous reports of resistance.

It was established that animals in the control group should receive salvage treatment when two situations occurred: (a) when the average number of ticks in the group was greater than 25, all animals were treated, or (b) when any animal had a tick count above 80, in which case treatment was individual and not for the whole group. However, during the observed period, it was not necessary to perform any salvage treatment.

The animals in the SC1S group received three treatments with the commercial formulation at 42-day intervals starting in early November, a period in which there was an increase in rainfall. The treatments occurred in spring/summer: day 0, 11 November 2020; day 42, 21 December 2020; and day 84, 03 February 2021.

On the same dates at the beginning of the rainy season, the animals in the SC2S group received treatments with 5% fluralaner. This group received another three treatments in the transition between the rainy season and the dry season on fall/winter, on day 182, 12 May 2021; day 224, 23 June 2021; and day 266, 04 August 2021.

In both treated groups, salvage treatments would be performed in periods outside those foreseen for strategic control, following the same criteria described above for the control group.

All animals were dewormed with levamisole (Ripercol^®^) at a dose of 1 mL/40 kg body weight on day 0. Throughout the experimental period, they were monitored fortnightly for the presence of gastrointestinal nematodes by means of fecal egg count. This treatment was performed two more times, on day 182 and day 280.

### Analytical procedures

The counts of semi-engorged female *R. microplus* measuring between 4.5 and 8.0 mm [21] were performed only on the left side of the animals, always between 8:00 and 10:00 a.m. and by the same person using a measuring plate. To perform the tick counts, the animals were physically restrained in a containment trunk. At that time, the animals were also weighed without the need for prior fasting.

Considering the day of the first treatment as day 0, tick counts were performed, and animals were weighed at 14-day intervals until the end of the study on experimental day 336. All animals were subjected to semi-engorged female ticks counts 3 days after the first treatment and 7 days after each of the six treatments, in addition to counting at 14-day intervals evaluate drug efficacy.

### Acaricide efficacy

The therapeutic percentages and residual efficacy of fluralaner were calculated on the basis of the average tick counts performed on one side of each animal’s body, according to the formula recommended by Ministry of Agriculture, Livestock, and Food Supply (MAPA), Secretariat for Agricultural Defense (SDA), Ordinance no. 48, 12/05/1997: Efficacy = [1 − (Ta × Cb/Tb × Ca)] × 100, where:

Ta is the average number of ticks counted in the post-treatment period in animals of both treated groups until day 182; from that experimental day, the average number of ticks was counted only in the SC2S group.

Tb is the mean number of ticks counted on day 0 in animals of both treated groups until day 182; from that experimental day, the average number of ticks was counted on day 0 only in the SC2S group.

Ca is the mean number of ticks counted in the post-treatment period on animals in the negative control group.

Cb is the mean number of ticks counted on day 0 on animals in the negative control group.

### Climatic data

A complete digital weather station (Instrutemp—ITWH-1080) was installed at the study site to measure temperature, relative air humidity (RAH), and precipitation, with measurements recorded every 30 min. The minimum and maximum temperature and humidity, as well as the daily average, were obtained from the data collected by the meteorological station, and the averages for each day on which counting and weighing of the animals were performed were then calculated. Accumulated rainfall data were obtained from the sum of the precipitation (mm) at each interval between tick counts.

### Return on investment (ROI) comparative analysis

Return on Investment (ROI) was applied to compare the different treatment groups. Thus, we calculated the return on funds invested in animals subjected to the SC1S compared with the SC2S protocol.

Prior to the ROI comparative analysis, the following values were determined for each treatment protocol: cost of ectoparasiticide treatment (CET), gross profit per animal (GPA), and net profit per animal (NPA). All metrics were calculated per individual, considering the average of the total number of animals included in each group (*n* = 12). The results were expressed in dollars (US$).

The cost of ectoparasiticide treatment (CET) was calculated by adding the average cost of each treatment for protocols SC1S (03 treatments) and SC2S (06 treatments) as follows: CET = ∑ Average dose used on each experimental day × (cost of the antiparasitic/total volume of the commercial product bottle). For the GPA simulation, the pricing of crossbred heifers (Nelore × Angus) in the 18–24 month age range determined on the basis of live weight based on the Center for Advanced Studies in Applied Economics (CEPE; Esalq/USP, Brazil) official database was considered. At the end of the experiment (October/2021), the price charged for the animal category evaluated was US$1.97 per live kg. In this way, the GPA was calculated by multiplying the mean final GPV (day 322) of each group and the live kg value found at the end of the experiment (IR = final GPV × live Kg value). After determining the CET and GPA, the estimated gross profit (EGP) for each protocol was calculated (NPA = GPA − CET).

Following the methodology used by Gomes et al. [22], the comparative analysis of the return on invested resources (ROI) between the two treatments was carried out by dividing the NPA differential of the SC2S and SC1S protocols by the CET differential of the SC2S and SC1S protocols (ROI = NPA differential ÷ differential of CET).

### Statistical analysis

A split-plot in time design was used, considering treatments to be the main plot and the observation dates to be the secondary plot. The D’Agostino–Person test was applied to evaluate the normality of the individual data of tick counts and weights of the animals, and then, two-way analysis of variance, treatment, and time, with repeated measures in the time factor (two-way ANOVA MR), followed by Sidak’s test of multiple comparisons was used to verify differences between groups as well as differences within each group on the different evaluation dates at the 95% significance level.

Pearson’s correlation coefficient was used to evaluate the relationship between the climatic variables: relative humidity, rainfall, average temperature, minimum temperature, and maximum temperature, and the average number of ticks after 56 days, considering the time required from the detachment of mature females, through development in the environment, to the parasitic stage, and ending with the partially engorged female stage [19].

To study the seasonality of semi-engorged female ticks in the animals in the control group, analysis of variance (ANOVA) was performed, and then the results for each date were compared with those for the date with the lowest mean. The means that were higher than the lowest mean according to Dunnett’s test of multiple comparisons were considered peaks.

All analyses were performed using GraphPad Prism version 6.0 software for Windows (GraphPad Software, San Diego, California, USA).

## Results

**Fig. 1:**
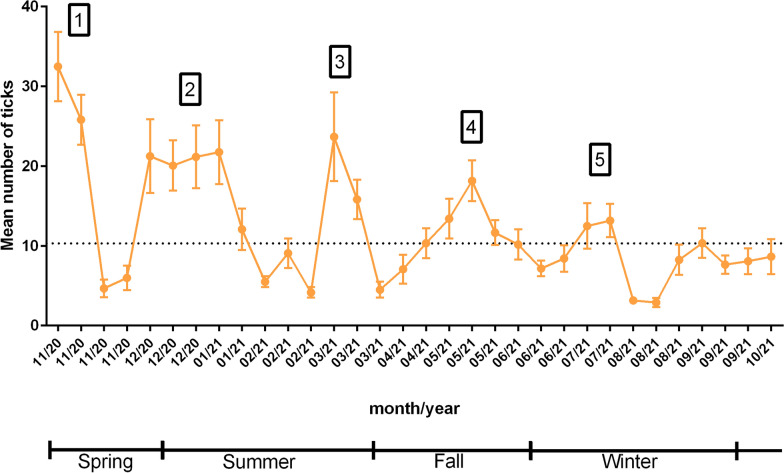
Means and standard errors of the number of ticks on each counting day for the animals in the control group. The dotted line represents the threshold of 10.3 ticks, which is the highest mean that did not differ from the lowest mean of the control group

During the experimental period, five peaks of tick infestation were identified in the animals in the control group (Fig. 1). The cutoff point for determining an infestation peak was the highest mean (i.e., 10.3 ticks) that did not differ (two-way ANOVA, *F*_(62;,1023)_ = 8.513; *P* > 0.05) from the lowest mean observed. When the mean counts were higher than 10.3 ticks, we considered this to be an infestation peak.

The first peak of infestation occurred in spring, at the beginning of the rainy season and the experiment, in November 2020 on day 0 and day 3. Two more peaks were observed during the summer, in December 2020, on day 28, day 40, day 48, and day 56, and in March 2021, day 126; one was observed in the autumn, day 189 in May 2021; and one was observed in the winter, day 252 in July 2021, when there was less rainfall.

**Fig. 2:**
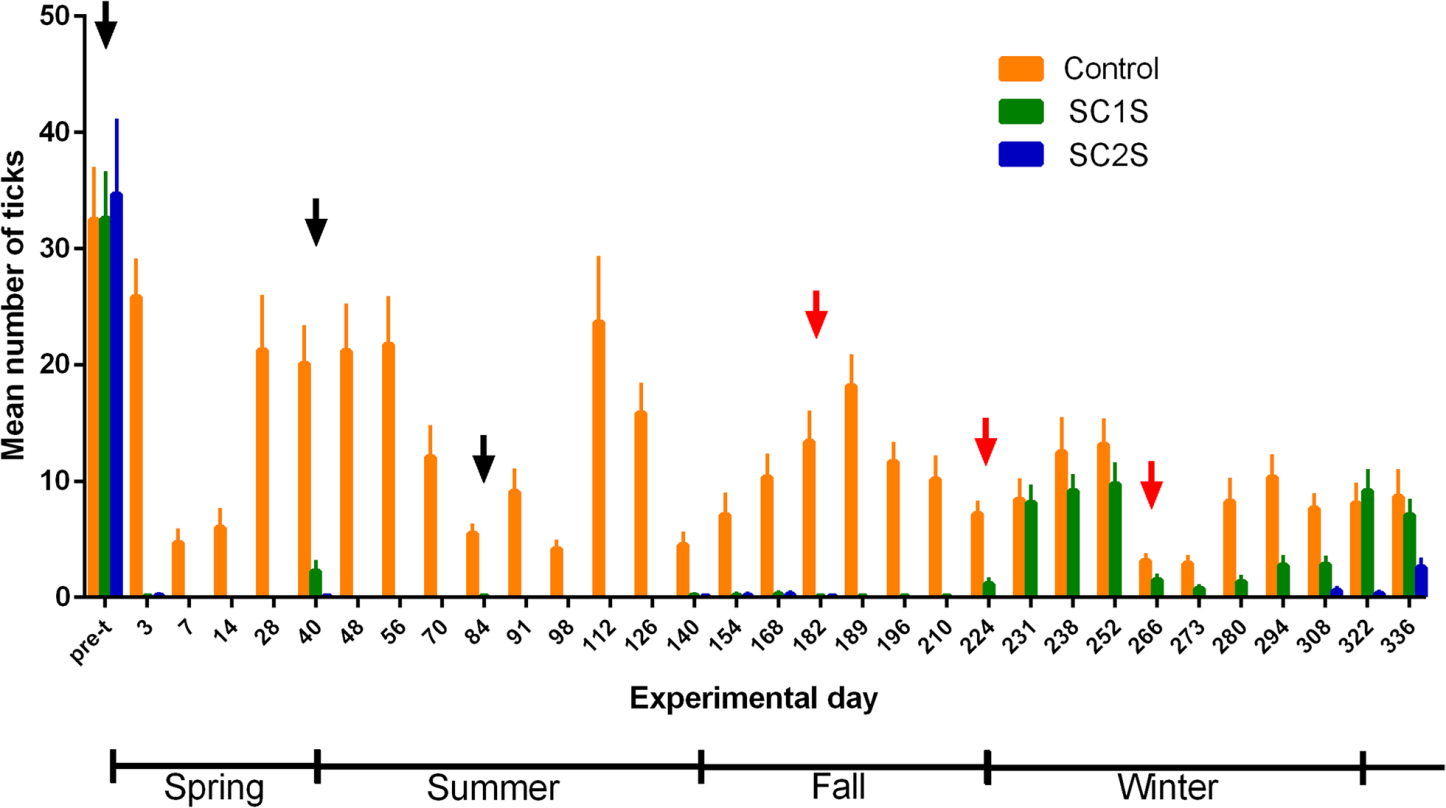
Means and standard errors of the number of ticks on each counting day for the animals in the three experimental groups. The black arrows indicate treatments in animals from the SC1S and SC2S groups, and the red arrows indicate treatments only in animals from the SC2S group

The two groups that received treatment showed a decrease in mean counts on the third day after the first treatment, and the means remained low throughout the experimental period for the SC2S group (Fig. 2). In the SC1S group, there was an increase in the average tick counts during winter of 2021 on day 231, with averages similar to those observed in the control group (Table 1). These results indicate that the tick population was susceptible to fluazuron.

**Table 1:** Means and standard errors of the tick counts for each experimental day and the mean efficacy of 5% fluralaner on traditional strategic control in a single season (SC1S), at the beginning of the rainy season, and strategic control in two seasons (SC2S), at the beginning and end of the rainy season

Day	Control group			SC1S			SC2S			Mean efficacy of fluralaner 5% (%)	DAT
	Mean	SEM		Mean	SEM		Mean	SEM			
Pre	32.50	4.36	a	32.58	3.93	a	34.67	6.37	a		
3	25.83	3.15	a	0.08	0.08	b	0.17	0.11	b	99.5	3
7	4.67	1.12	a	0.00	0.00	a	0.00	0.00	a	100.0	7
14	6.00	1.54	a	0.00	0.00	b	0.00	0.00	b	100.0	14
28	21.25	4.62	a	0.00	0.00	b	0.00	0.00	b	100.0	28
40	20.08	3.16	a	2.25	0.84	b	0.08	0.08	b	94.2	40
48	21.17	3.95	a	0.00	0.00	b	0.00	0.00	b	100.0	8
56	21.75	4.00	a	0.00	0.00	b	0.00	0.00	b	100.0	16
70	12.08	2.60	a	0.00	0.00	b	0.00	0.00	b	100.0	30
84	5.50	0.69	a	0.08	0.08	a	0.00	0.00	a	99.2	44
91	9.08	1.85	a	0.00	0.00	b	0.00	0.00	b	100.0	7
98	4.17	0.67	a	0.00	0.00	a	0.00	0.00	a	100.0	14
112	23.67	5.56	a	0.00	0.00	b	0.00	0.00	b	100.0	28
126	15.83	2.47	a	0.00	0.00	b	0.00	0.00	b	100.0	42
140	4.50	1.00	a	0.17	0.11	a	0.08	0.08	a	97.2	56
154	7.08	1.81	a	0.17	0.17	b	0.17	0.17	b	97.6	70
168	10.33	1.89	a	0.25	0.18	b	0.25	0.18	b	97.6	84
182	13.42	2.49	a	0.08	0.08	b	0.08	0.08	b	99.4	98
189	18.17	2.57	a	0.08	0.08	b	0.00	0.00	b	100.0	7**
196	11.67	1.56	a	0.08	0.08	b	0.00	0.00	b	100.0	14
210	10.17	1.89	a	0.08	0.08	b	0.00	0.00	b	100.0	28
224	7.17	0.98	a	1.17	0.39	b	0.00	0.00	b	100.0	42
231	8.42	1.66	a	8.17	1.38	a	0.00	0.00	b	100.0	7
238	12.50	2.86	a	9.17	1.32	a	0.00	0.00	b	100.0	14
252	13.17	2.10	a	9.75	1.73	a	0.00	0.00	b	100.0	28
266	3.17	0.49	a	1.50	0.38	a	0.00	0.00	a	100.0	42
273	2.92	0.58	a	0.75	0.25	a	0.00	0.00	a	100.0	7
280	8.25	1.90	a	1.33	0.45	b	0.00	0.00	b	100.0	14
294	10.33	1.85	a	2.75	0.75	b	0.00	0.00	b	100.0	28
308	7.67	1.16	a	2.83	0.63	ab	0.58	0.23	b	92.4	42
322	8.08	1.63	a	9.17	1.71	a	0.33	0.19	b	95.9	56
336	8.67	2.20	a	7.08	1.27	ab	2.58	0.71	b	70.2	70

Different lowercase letters on the same line represent significant differences in the mean tick count between experimental groups. Efficacy was calculated using the average number of ticks counted in the post-treatment period on animals of both treated groups until day 182; from that experimental day onward, the average number of ticks was counted in only the SC2S group. *SEM* standard error of the mean, *DAT* days after treatment

In the SC2S the efficacy of 5% fluralaner remained above 95% up to day 294, except for day 40 with 94.2% (Table 1). From day 308, the efficacy of fluralaner 5% decreased, reaching 70.2% on day 336, the lowest efficacy observed during the experimental period; however, this value was observed on the 70th day after the last treatment.

**Fig. 3:**
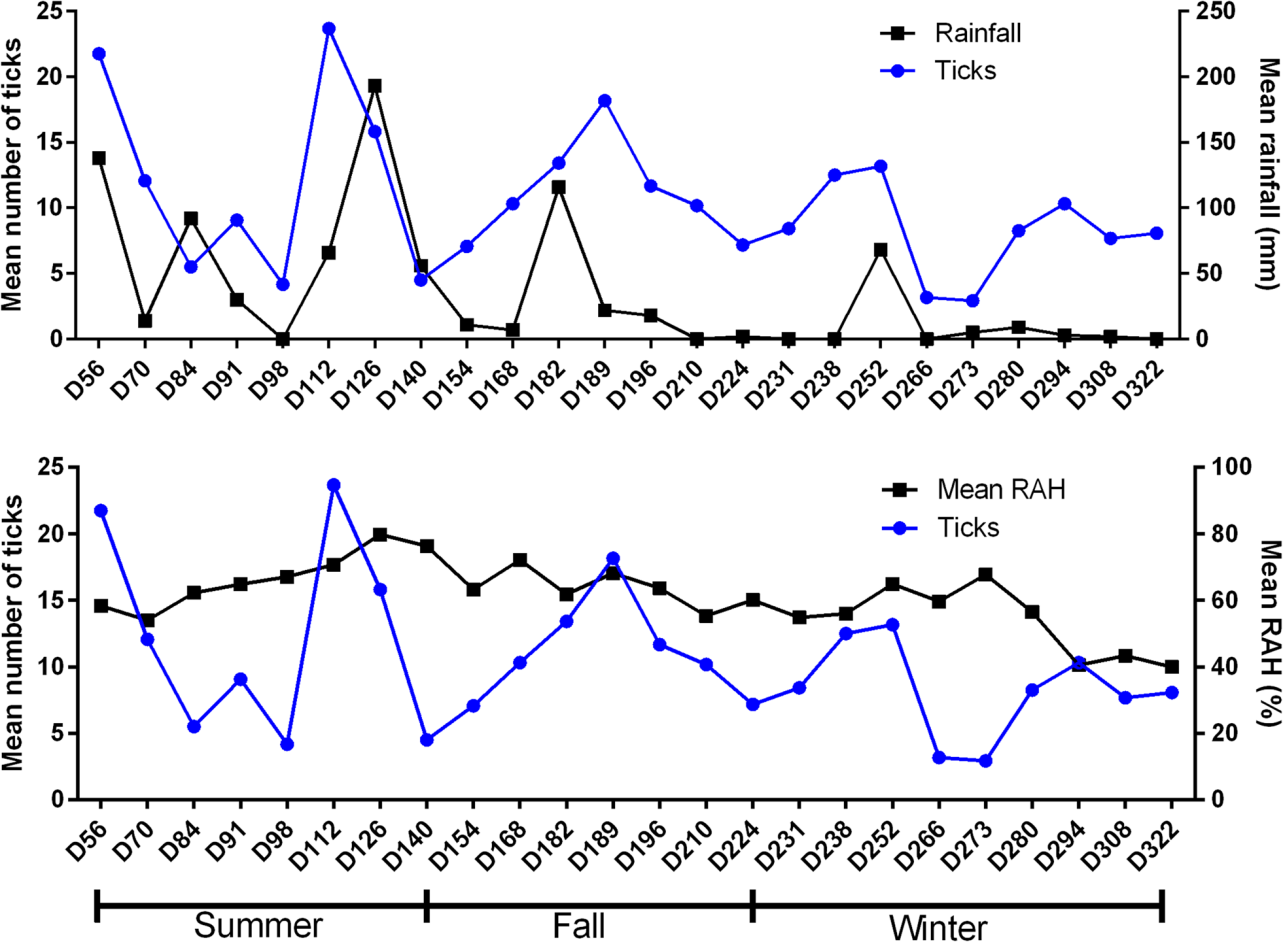
Accumulated rainfall volume (mm) in the interval before each count and the average tick count for the control group; graph of the average RAH for the period before each count and the average tick count for the control group

Beginning in April 2021 in mid-autumn, there was a reduction in the accumulated rainfall volume. From June 2021 to September 2021, in the winter, the accumulated rainfall volume was close to 0 mm, which also contributed to a reduction in the average RAH (Fig. 3).

**Fig. 4:**
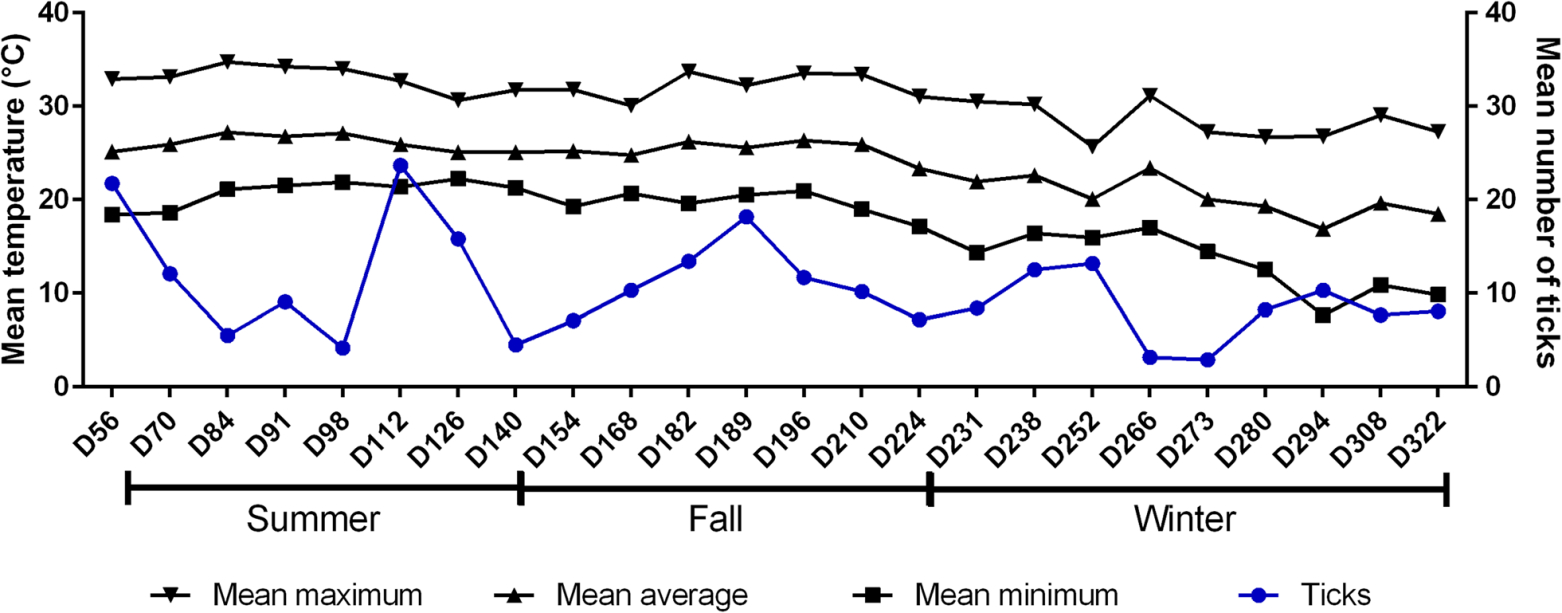
Mean values of the minimum, average, and maximum temperatures during the experimental period and the average tick counts in the control group. The red arrow indicates the period with the occurrence of frost

A decrease in the average minimum temperature was observed in June and July 2021, with frost occurring on days with minimum temperatures of 3.2 °C, 2.8 °C, and 1.2 °C on the 29th and 30th of June 2021 and the 1st of July 2021, respectively; these were the lowest temperatures recorded during the experiment. The average and maximum temperatures during the experimental period remained constant throughout the experimental period (Fig. 4).

The lowest mean tick counts in the control group occurred in the same period in which the lowest accumulated rainfall was observed, the lowest mean RAH occurred in the period with the lowest average minimum temperature, and the lowest counts occurred approximately 30–45 days after the frosts on day 266, with mean tick number 3.17, and day 273 = 2.92.

Pearson’s correlation coefficients between the climatic variables and the mean tick count after 56 days in the control group revealed that the only climate variable that showed a significant positive correlation (Pearson’s correlation coefficient, *r*_(24)_ = 0.516; *P* = 0.01) was rainfall (Table 2). However, this result does not rule out the effect of frost on the tick averages described above.

**Table 2:** Pearson’s correlation for climatic variables and the mean tick count in the control group

Climatic variable	Mean number of ticks in the control group	
	*r*	*P*-value
Rainfall	0.516	0.01
Relative air humidity	0.149	0.486
Minimum temperature	0.233	0.274
Average temperature	0.213	0.317
Maximum temperature	0.160	0.454

**Fig. 5:**
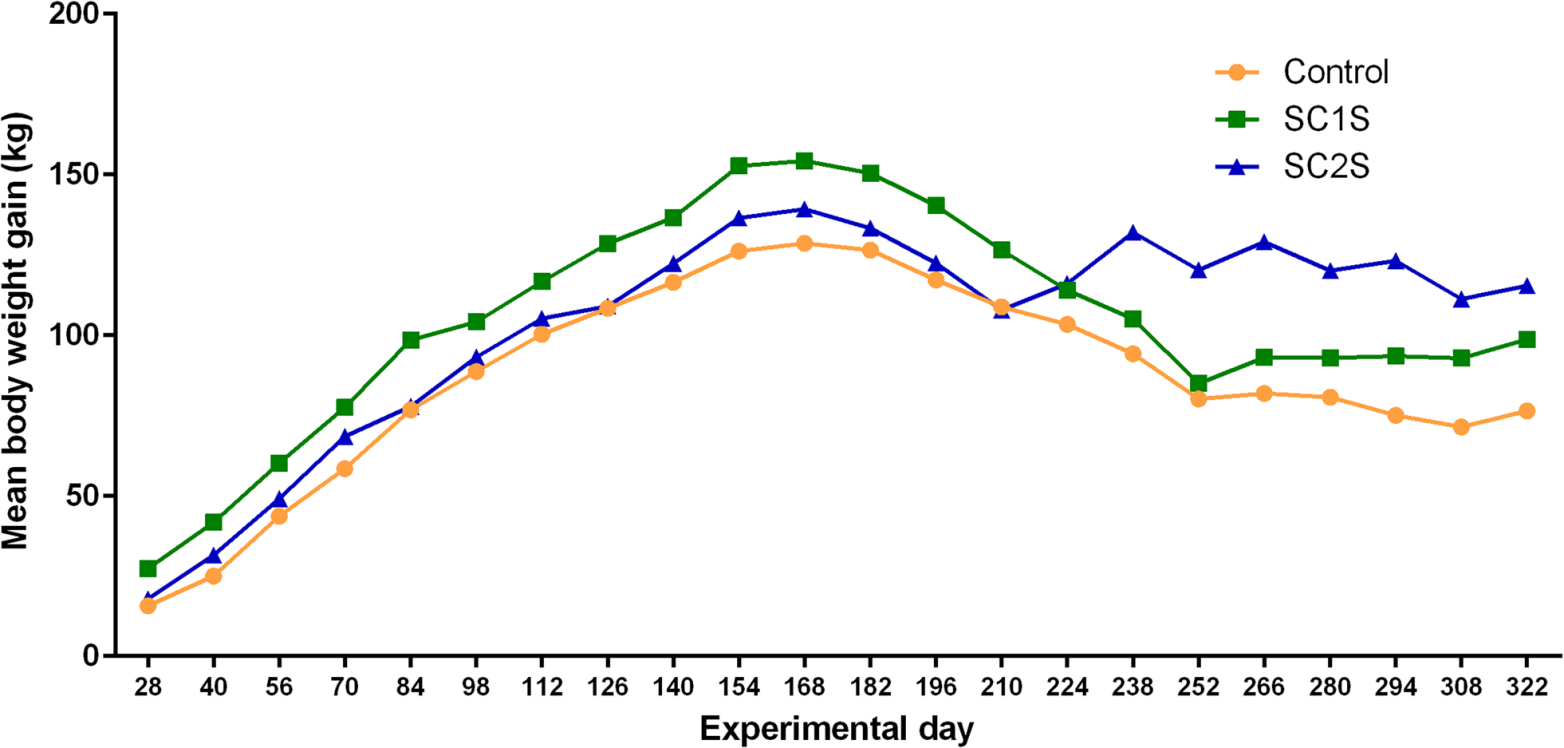
Body weight gain of the SC1S, SC2S, and control groups between day 28 and day 322

There was a difference (two-way ANOVA, *F*_(2,33)_ = 11.74; *P* = 0.0001) in final weight gain on day 322 among the three groups, with mean values of 115.38 kg, 98.63 kg, and 76.40 kg for the SC2S, SC1S, and control groups, respectively (Fig. 5 and Table 3). Although the animals received protein-energy supplementation, all experimental groups showed weight loss starting on day 168, May 2021, due to the decrease in pasture supply with the onset of the drought period.

**Table 3:** Mean body weight and mean body weight gain (BWG) of the control group, SC1S, and SC2S, evaluated between day −2 and day 322

Day	Control				SC1S				SC2S			
	Weight (kg)	BWG (kg)	SEM		Weight (kg)	BWG (kg)	SEM		Weight (kg)	BWG (kg)	SEM	
−2	167.7				168.0				181.0			
28	183.5	15.75	2.18	a	195.4	27.33	2.62	b	198.8	17.83	1.75	a, b
40	192.7	25.00	2.22	a	209.7	41.67	3.07	b	212.4	31.42	2.16	a, b
56	211.3	43.63	2.47	a	228.2	60.13	2.25	b	230.0	49.00	1.54	a, b
70	226.1	58.38	2.66	a	245.5	77.46	2.73	b	249.3	68.38	1.93	a, b
84	244.4	76.71	2.53	a	266.5	98.46	3.41	b	258.8	77.79	1.59	a
98	256.3	88.63	2.85	a	272.2	104.13	3.73	b	274.1	93.13	1.76	a, b
112	268.0	100.29	3.21	a	284.7	116.63	3.60	b	286.1	105.13	2.15	a
126	276.1	108.38	3.40	a	296.5	128.46	4.01	b	290.0	109.04	2.24	a
140	284.2	116.46	3.58	a	304.6	136.54	4.55	b	303.3	122.29	2.60	a
154	293.8	126.13	3.96	a	320.8	152.71	4.90	b	317.4	136.46	3.96	a
168	296.3	128.63	3.90	a	322.3	154.21	4.27	b	320.2	139.21	3.66	a
182	294.2	126.46	3.76	a	318.4	150.38	4.63	b	314.3	133.29	3.79	a
196	284.9	117.21	3.85	a	308.3	140.29	4.22	b	303.4	122.46	3.42	a
210	276.5	108.79	3.92	a	294.5	126.46	3.24	b	288.8	107.88	2.90	a
224	271.1	103.38	3.63	a	282.1	114.04	3.94	ab	297.0	116.04	3.01	b
238	261.9	94.21	3.82	a	273.1	105.04	4.64	a	313.0	132.04	3.40	b
252	247.8	80.13	3.48	a	253.0	84.96	3.68	a	301.3	120.29	3.68	b
266	249.5	81.79	3.76	a	261.1	93.04	4.08	b	310.0	129.04	3.42	c
280	248.3	80.63	3.85	a	261.0	92.96	3.46	b	301.1	120.13	3.10	c
294	242.8	75.04	3.93	a	261.5	93.46	3.69	b	304.2	123.21	3.84	c
308	239.2	71.46	3.24	a	260.8	92.79	3.10	b	292.2	111.21	2.93	c
322	244.1	76.38	3.50	a	266.7	98.63	3.94	b	296.3	115.38	2.87	c

Different lowercase letters on the same line represent significant differences in mean weight gain according to Tukey’s multiple comparison test

**Fig. 6:**
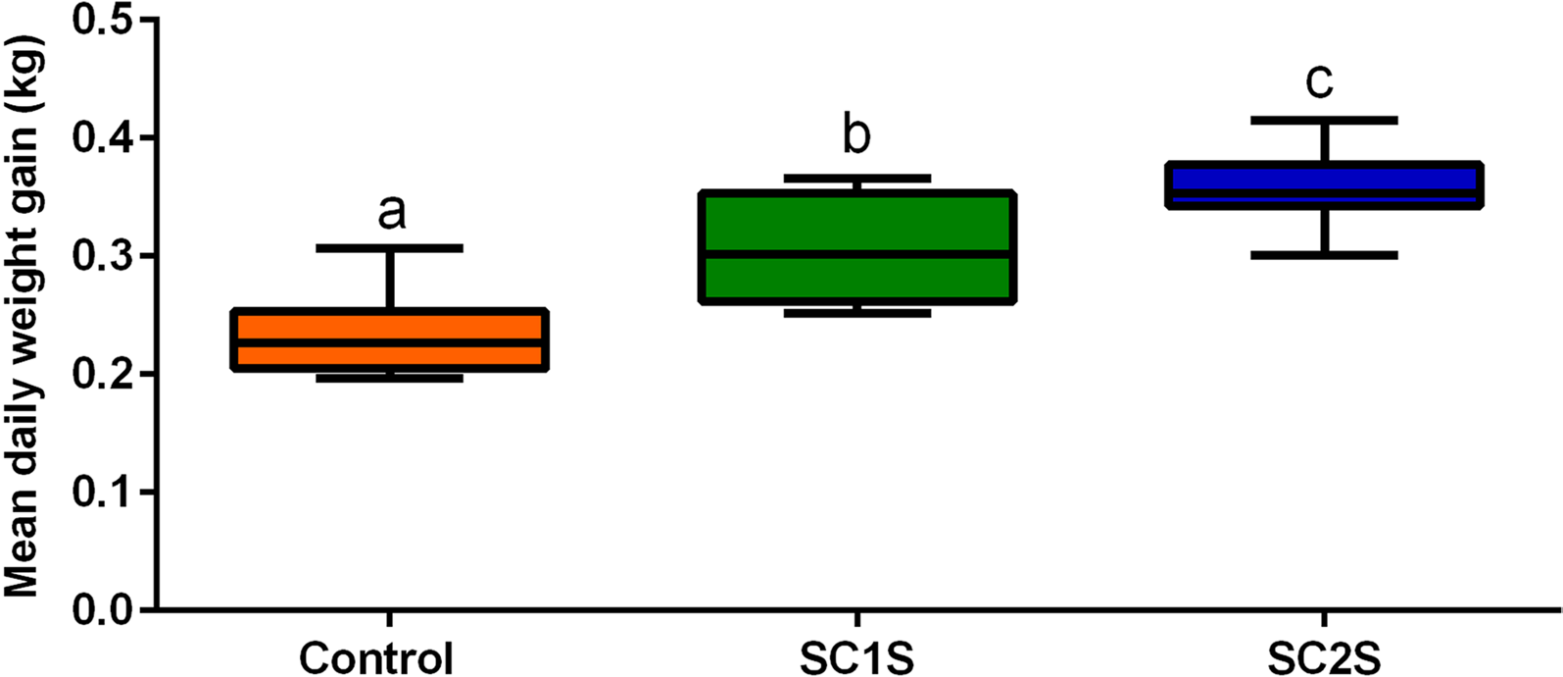
Boxplot showing the quartile (1st, 2nd, 3rd, and 4th) and median final mean daily weight gains of Nellore × Angus heifers subjected to two tick control protocols with fluralaner and in the control group. Different letters signify significant differences according to Tukey’s test of multiple comparisons

The animals in the SC2S group had a mean daily gain (MDG) of 0.36 kg on day 322 (Fig. 6), which was significantly higher (Tukey’s multiple comparisons test, *q* = 11.31, *df* = 33; *P* < 0.0001) than that in the control group, which had a MDG of 0.24 kg. The MDG of the SC1S group fell between those of the control group and the SC2S group at 0.31 kg; this value was significantly different from those for both the control (Tukey’s multiple comparisons test, *q* = 6.469, *df* = 33; *P* = 0.0002) and SC2S (*Tukey’s multiple comparisons test, q* = *4.840, df* = *33, P* = 0.0046) groups.

The result of the comparative ROI analyzes for the different treatment protocols (SC2S × SC1S) are presented in Table 4. The SC2S protocol increased the treatment cost by 2.44 times in relation to the SC1S protocol. In contrast, the ROI of animals subjected to SC2S was greater (ROI = 0.34) than animals in the SC1S group.

**Table 4:** Cost of Ectoparasiticide Treatment (CET), Gross Profit per Animal (GPA), Net Profit per Animal (NPA) and Return on Investment (ROI) comparative analysis for traditional strategic control in a single season (SC1S), and strategic control in two seasons (SC2S) groups

CET (US$)	
SC2S	$41.66
SC1S	$17.06
Cost differential (SC2S − SC1S)	$24.60

GPA (US$)	
SC2S	$227.00
SC1S	$194.05
Differential GPA (SC2S − SC1S)	$32.95

NPA (US$)	
SC2S	$185.34
SC1S	$176.99
Differential NPA (SC2C − SC1C)	$8.35

Comparative ROI	
ROI (SC2S/SC1C)	0.34

Estimated values are in US dollars. *GPA* CET gross profit per animal, *NPA* net profit per animal, and *ROI* return on investment

## Discussion

This controlled and randomized clinical trial demonstrated the efficiency of fluralaner in the strategic control of cattle ticks under tropical conditions. It also compared the strategic control scheme traditionally recommended for the study region (i.e., winter–spring strategic control) [17, 18] with the most recent recommendation for treatment in two seasons (i.e., winter–spring and autumn–winter strategic control), on the basis of more recent data about tick seasonality in tropical and subtropical areas [19, 20].

During the experimental period, the study region experienced a period of severe drought, which reduced pasture forage and consequently the weight gain of the animals, resulting in weight loss from day 168 onward. This drought period may also have negatively affected the environmental challenge of ticks to which the animals were exposed, with a reduction in tick infestation in the control group.

In the control group, we documented five peaks of infestation in a period of 1 year, which deviates from the pattern expected for the geographical region, which would be three to four peaks [12–14]. Gomes et al. [23], Cruz et al. [20], and Nicaretta et al. [19], in studies evaluating the population dynamics of *R. microplus* in areas of the Brazilian cerrado in Minas Gerais, São Paulo, and Goiás, respectively, reported a greater number of infestation peaks, with the occurrence of new peaks during the period that comprises the winter, which corroborates the findings of the present study. In northern Argentina, which is characterized as subtropical, an increase in the number of peaks of tick infestation was also observed [16]. A possible reason for the occurrence of an additional peak in the winter period is the increase in temperature observed in the last 30 years [19]. However, in the present study, the winter was dry, with minimum temperatures close to 1 °C and the occurrence of frosts.

High summer temperatures lead to an increase in the number of ticks, favoring a higher infestation peak during the autumn [13, 14, 18–20, 22]. This pattern differs from that observed in the present study, in which a trend of reduction in the intensity of the peaks was observed, which suggests the development of immunity of animals to ticks. These results are similar to the findings of a study by Cruz et al. [20], in which crossbred animals were used for two consecutive years, and the peaks observed in the second year were lower than those in the first year, and the findings of a study by Martins et al. [24], in which the autumn peak was lower than the peaks observed in the summer in crossbred animals.

The strategic control protocol recommended for the Brazilian cerrado provides for the treatment of animals with acaricides from late winter, the beginning of the rainy season, to mid-spring, with the interval between and number of treatments determined according to the molecule used in the treatments [17]. In the present study, the winter–spring strategic control protocol resulted in control of tick infestations until the end of the rainy season and the beginning of the dry season, day 224, on 23 June 2021, as observed in other studies [14, 18].

Approximately 150 days after the last treatment, on day 231, the SC1S group, which was subjected to the winter–spring strategic control protocol, presented a level of infestation similar to that observed in the control group. On the basis of this finding, it was suggested that, in tropical and subtropical regions, when conditions allow the occurrence of a peak tick infestation during the winter, a new round of treatments to control *R. microplus* during this season could be justified. This strategies control approach was implemented in the SC2S group, with treatments being performed both at the time of the winter–spring strategic control protocol and at the end of the rainy season and beginning of the dry season, i.e., during late autumn and the beginning of winter. This approach should make it possible to control tick infestations in cattle throughout the year and allow for greater weight gain by cattle.

In a cattle herd, located in the same region as the present study, with a population of ticks resistant to multiple drugs, treatments with fipronil, fluazuron, and moxidectin did not present satisfactory efficiency, and the only viable economic treatment was a spray combination of chlorpyrifos 30 g, cypermethrin 15 g, and fenthion 15 g [25]. Although this treatment was economically viable, this type of atomizing chamber treatment is not adopted on beef cattle farms in Brazil, and there is also the problem of the reduced residual period of spray treatments. Given the severity of *R. microplus* resistance to acaricides [4, 7, 26–31], a new molecule such as fluralaner may be one of the few chemical alternatives available. This isoxazoline showed efficacy above 95% as early as the third day after treatment. When applied three times at 42-day intervals, this strategy maintained high percentages of efficacy for approximately 150 days after the last treatment in the SC1S group, which was subjected to the winter–spring strategic control protocol, and for approximately 70 days after the last treatment in the SC2S group, which received winter–spring and autumn–winter strategic controls, thus controlling *R. microplus* throughout the experimental year.

With this new protocol, in addition to year-round tick control in this experimental group, greater weight gain was also observed compared with the control and SC1S groups, which corroborates the findings of Calvano et al. [32], demonstrating that effective tick control in cattle allows an increase in weight gain by these animals, increasing the productivity of herds intended mainly for meat production. According to the economic analysis carried out in this study, although the addition of three treatments at the end of the rainy season increased the treatment cost by 2.44 times, the return on investment of animals that were treated with ectoparasiticides at the beginning and end of the rainy season was higher (ROI = 0.34) than the return of animals that were treated only at the beginning of the rainy season. In this way, the financial returns of the two-season treatment protocol (SC2S) exceeded the costs and investment.

However, there are caveats when generalizing the data obtained in this study to all tropical regions, as in situations that favor greater challenges from ticks, two consequences may occur: the impact on weight gain may be much greater, and the residual period of the product may be shorter. Therefore, we point out the need for studies such as this in different climatic conditions, cattle breeds, and tick challenges.

## Conclusions

The winter–spring strategic control protocol with 5% fluralaner resulted in control of *R. microplus* for 224 days, throughout the rainy season and until the beginning of the dry season. However, to control a possible fifth peak of infestation, which occurs in the dry period, a second set of treatments could be recommended in the autumn–winter season, also resulting in greater weight gain among cattle.

## Data Availability

All data are included as tables and figures in the article.
